# Iatrogenic wounds: a common but often overlooked problem

**DOI:** 10.1186/s41038-019-0155-2

**Published:** 2019-06-01

**Authors:** Biao Cheng, Ju Tian, Yan Peng, Xiaobing Fu

**Affiliations:** 1 Department of Plastic Surgery, General Hospital of Southern Theater Command of PLA, 111 Liuhua Road, Guangzhou, 510010 Guangdong People’s Republic of China; 2 Department of Plastic Surgery and the Key Laboratory of Trauma Treatment and Tissue Repair of Tropical Area, PLA, Guangzhou, People’s Republic of China; 3 Department of Orthopaedics and Traumatology, Li Ka Shing Faculty of Medicine, The University of Hong Kong, 21 Sassoon Road, Pokulam, Hong Kong SAR People’s Republic of China; 4 Wound Healing Unit, The First Affiliated Hospital, General Hospital of PLA, 51 Fu Cheng Road, Beijing, 100048 People’s Republic of China; 5 Department of Plastic Surgery, Zhongshan City People’s Hospital, Zhongshan, 528400 Guangdong People’s Republic of China

**Keywords:** Wound, Iatrogenic, Implantable, Biomaterial, Surgical site infection

## Abstract

Iatrogenic wounds are a common but often overlooked concept. They can lead to increases in hospital stays, therapy costs, repeat surgeries, and implant removal. If not handled properly, these wounds have a very poor prognosis and will cause serious physical and psychological harm to patients, which may result in medicolegal disputes. In recent years, the incidence of iatrogenic wounds has increased because of (1) an increase in the population of older people owing to increased life expectancy, (2) the continued expansion of surgical indications, (3) an increase in difficult surgeries, and (4) the constant emergence and application of new implantable biomaterials and other therapies. Thus, there is a pressing clinical need to improve the therapy of iatrogenic wounds. However, the difficulty in treating these wounds is considerable due to the emergence of drug-resistant bacteria, the high number of patients with metabolic diseases, and complex complications in patients. In particular, iatrogenic wounds caused by surgical site infections due to implantable biomaterials could lead to material leakage and conflicts regarding whether to retain or remove the implants. This review provides a definition of iatrogenic wounds, describes their characteristics, classifies them, and provides information about the importance of analyzing iatrogenic wounds. We hope that this review will provide useful information for the diagnosis and treatment of iatrogenic wounds and help to reduce their incidence in the future.

## Background

Although the quality of medical and surgical care has improved remarkably, the incidence of iatrogenic wounds has been increasing in recent years [1]. Moreover, the treatment of some iatrogenic wounds is very difficult. Iatrogenic wounds can increase hospital stays and therapy costs and lead to repeat surgery and implant removal. If not handled properly, these wounds may have a very poor prognosis and cause serious physical and psychological harm to patients, which may lead to medical disputes. Thus far, there has been no systematic long-term analytical study of iatrogenic wounds. In this review, we aim to reveal the characteristics of iatrogenic wounds and hope to provide useful knowledge for their diagnosis and treatment.

## Review

### Definition of iatrogenic wounds

Iatrogenic injury refers to tissue or organ damage that is caused by necessary medical treatment, pharmacotherapy, or the application of medical devices and has nothing to do with the primary disease [2]. The definition of iatrogenic wounds is derived from iatrogenic injury. When the integrity of the skin, subcutaneous soft tissue, and even deep tissue is compromised, the resulting defect is termed an iatrogenic wound. Iatrogenic wounds include various acute wounds (e.g., skin donor site wound and injury due to laser treatment), complications resulting from various treatments and operations (e.g., surgical site infections (SSIs)), and chronic wounds caused by improper medical treatment (e.g., hospital-acquired pressure ulcers and radiation ulcers) [3–7]. Iatrogenic wounds can involve damage to the superficial tissues, such as the skin and soft tissues, or to the deep tissues, such as the bones and tendons. Thus, the term iatrogenic wounds is more extensive than the terms iatrogenic skin injury [8] and iatrogenic skin and soft tissue injury [9].

### Historical evolution of iatrogenic injury and iatrogenic wounds

**Fig. 1:**
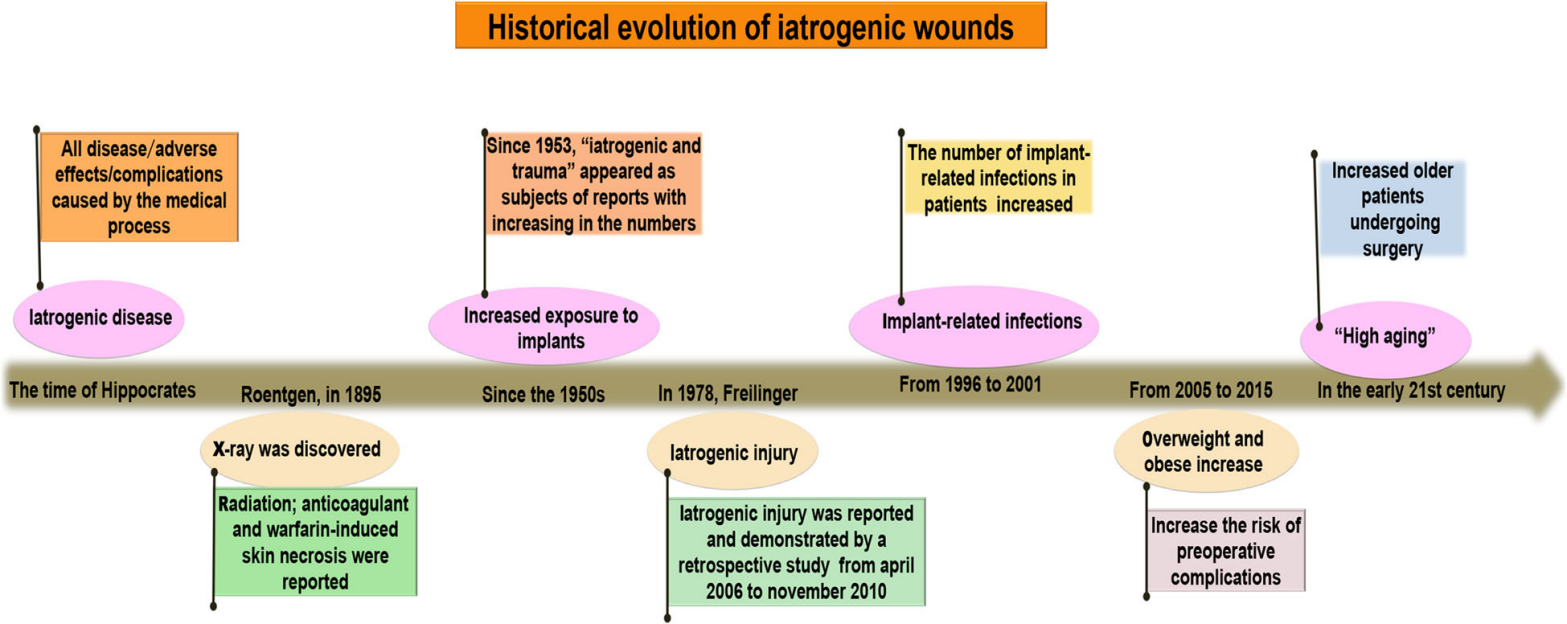
A brief historical evolution of iatrogenic wounds

The historical evolution of iatrogenic wounds is shown in Fig. 1 [5, 10–15]. The term iatrogenesis means “brought forth by a healer” and is derived from the Greek ἰατρός (iatros, “healer”) and γένεσις (genesis, “origin”), so it could refer to good or bad effects. Since at least the time of Hippocrates, people have recognized that a healer could cure diseases but also cause potential damage [10]. X-rays were discovered by Roentgen in 1895, and radiation-induced skin damage was reported the following year [11]. Stacher first reported skin necrosis due to anticoagulant therapy [12, 16]. Warfarin-induced skin necrosis almost always occurs by day 10 after therapy [17]. Since the 1950s, with the use of new biological materials, exposure to implants has increased [13]. Since 1953, the terms “iatrogenic” and “trauma” have appeared in an increasing number of reports [14]. In recent years, the incidence of iatrogenic wounds has increased [1]. The reasons for this increase are as follows [15, 18–21]: (1) human life has gradually prolonged, due to which the population of elderly people is increasing; (2) metabolic diseases are becoming increasingly more common; (3) surgical indications are expanding, more difficult operations are being performed, and operation time is getting longer; (4) new drugs (e.g., antitumor treatments, immune treatments, and hormones) and various types of implantable biological materials are being used; (5) drug-resistant bacteria have emerged; and (6) new therapeutic modalities, such as those involving electricity, magnetism, and light, are being developed.

### Characteristics of iatrogenic wounds

Iatrogenic wounds come under the purview of all hospital departments. Subcutaneous and deep artificial implants can result in wounds, so the incidence of iatrogenic wounds is increasing in surgical departments, especially in the fields of cardiac surgery, neurosurgery, orthopedics, and plastic surgery, which have witnessed the extensive use of biological materials, an expansion of surgical indications, and continual increases in the age limit for surgery [22, 23]. Surgeries for infants and young children are also becoming more common. Despite the advancements in medical science, the incidence of sternum infection and dehiscence after thoracotomy has remained unchanged. Skin and soft tissue necroses usually occur due to improper dressing, radiation therapy, or infusion treatment in oncology. The “cupping” therapy of traditional Chinese medicine and external treatment with herbs can lead to accidental burns and refractory wounds [24].

The consequences of some iatrogenic wounds are serious. If these wounds are not treated correctly, they may cause serious consequences and even death. For example, wounds caused by surgical implants may not show signs of infection even though many bacteria breed around surgical implants and present a clone-like growth pattern, and can even cause death.

### Classification of iatrogenic wounds

Like all wounds, iatrogenic wounds can be classified into acute, chronic, and refractory wounds, depending on the duration of the wound. According to the level of difficulty of treatment, iatrogenic wounds can be divided into simple wounds and complex or refractory wounds [25–27]. Additionally, iatrogenic wounds can also be divided into clean wounds, clean-contaminated wounds, contaminated wounds, and infected wounds.

The causes of some iatrogenic wounds are clearly defined and can be easily identified, such as complications of surgery. However, there exist also some less obvious iatrogenic wounds, such as wounds caused by complex drug interactions, which may be identified through careful and detailed research.

Iatrogenic wounds can also be divided into avoidable and unavoidable wounds. Unavoidable iatrogenic wounds are necessarily caused by the treatment itself, such as secondary wounds of postoperative laser stripping treatment for pigment disease and donor site wounds after skin and flap grafting in plastic surgery. Clean surgical wounds tend to heal without complications. Avoidable iatrogenic wounds include various interventions in medical practice, like implants or materials, side effects of drugs, and medical errors.

Iatrogenic wounds are not caused only by surgeons but can be caused by almost any healthcare professional, including physical therapists, radiation technicians, dermatologists, community doctors, laser therapists, and nurses. Furthermore, iatrogenic wounds are not associated with only modern medicine (e.g., implants, radiation meters, and electric knives), but can result from traditional medicine as well (e.g., topical traditional Chinese medicines, cupping, and moxibustion). Iatrogenic wounds can be caused by the increased use of new tissue substitutes, new photoelectric instruments, and new chemotherapy drugs as well as the expanding indications for treatments (e.g., increased patient-age range and basic diseases such as diabetes/high blood pressure control).

The classification of iatrogenic wounds is different from that of other wounds dependent on the cause of the pathogenic factors. According to the pathogenic factors involved, iatrogenic wounds may be divided into wounds caused by SSIs; wounds caused by radioactive damage; wounds caused by lasers, electric coagulation, or electric knives; and wounds caused by drugs. SSIs represent the second most common cause of hospital-acquired infections and the most common type of healthcare-associated infection and substantially contribute to annual morbidity, healthcare costs, and mortality [28–31]. Iatrogenic wounds caused by SSIs are often difficult to treat.

**Fig. 2:**
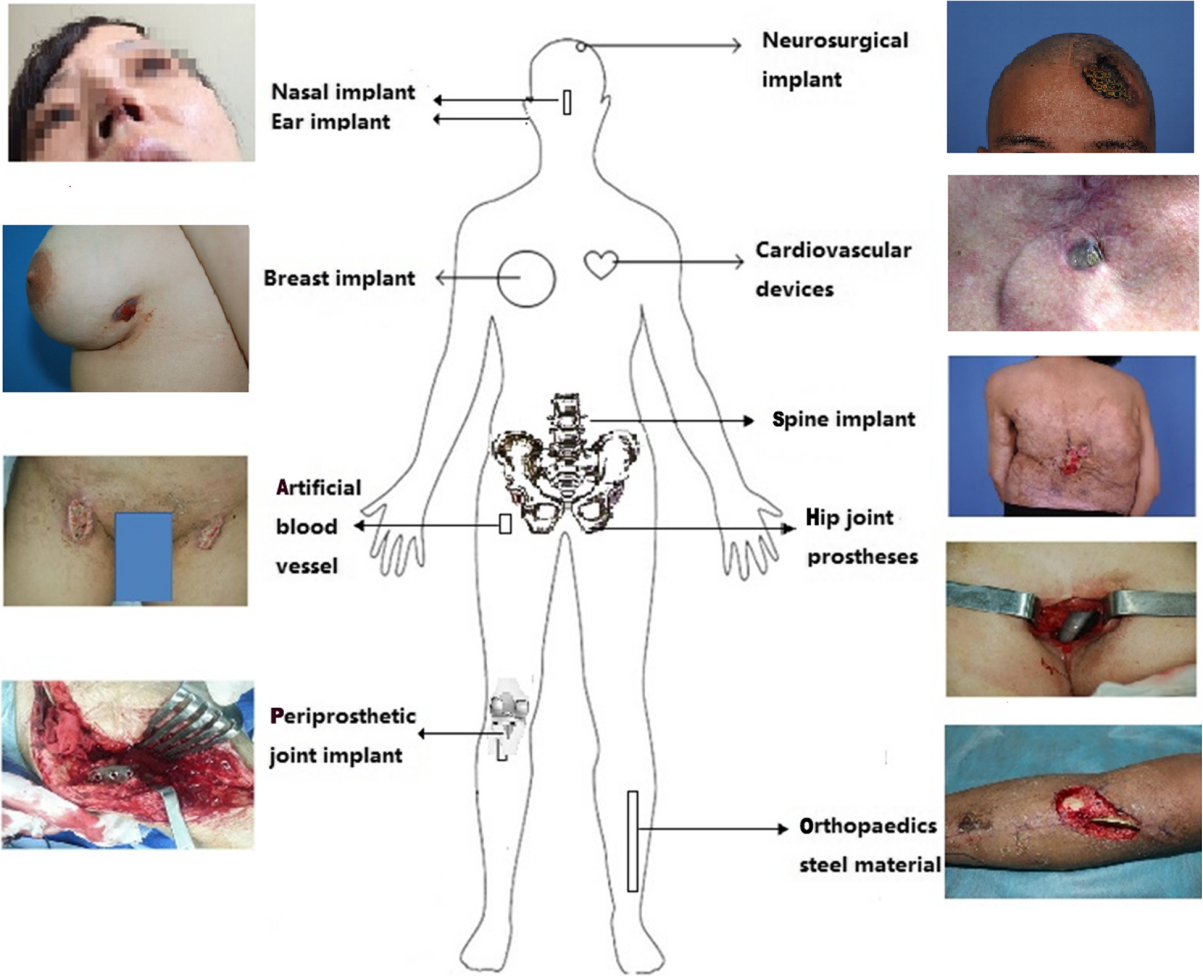
Iatrogenic wounds caused by surgical wound infection after implanted materials are implanted in different parts of the body

In recent years, wounds induced by implanted materials are the most common type of iatrogenic wounds, and these tend to be intractable (Fig. 2) [28–34]. It is estimated that the annual rate of infections associated with surgical implants could be close to one million [35]. Overall, 2.6 million patients receive orthopedic prostheses in the USA each year, and the number of infections related to orthopedic prostheses is close to 112,000 (about 4.3%) [36]. The infection rate after joint replacement is 1% to 10%, depending on the surgery type and technique employed, body location, and aftercare [37–39]. Vascular surgery and groin surgery are associated with a high rate of SSIs [40]. Moreover, biofilm-related infections caused by *Staphylococcus aureus* are increasingly being detected in patients receiving intravascular catheters, cardiac pacemakers, vascular grafts, mechanical heart valves, and orthopedic implants [41, 42].

The number of patients with cancer has been increasing sharply worldwide each year [43]. Many cancer patients need radiotherapy, and the radiation time and dose are also increasing. Although accurately estimating radioactive damage rates is difficult, the incidence of iatrogenic radioactive skin wounds has increased significantly.

### Prevention of iatrogenic wounds

Attention must be paid to pre-existing diseases, surgical time, wound contamination, patient age, malignant tumors, metabolic disease, malnutrition, immune suppression, smoking, etc. [44]. During surgery, frequent changes in the patient’s position must be reduced. Reasonable application of surgical instruments must be ensured, and we should increase antimicrobial treatment appropriately for patients with longer operation times or excessive blood loss. Changes in the patient’s position should be gentle during surgery to avoid damage to tissue. Radical removal of necrotic tissues in surgical sites must be ensured to prevent the formation of dead space. Close monitoring of body temperature is necessary during surgery to avoid temperature anomalies. Adequate intake of nutrients for patients must be ensured. The technique of fractionation of doses is used to minimize the risk of injury to normal tissue during radiation treatments [45]. Wound healing monitoring is an important concern in all surgical procedures since it allows the identification of signs or/and symptoms possibly related to surgical complications [46].

### Treatment of iatrogenic wounds

The principles of treatment of iatrogenic wounds are the same as those for other wounds, though the former do have their own unique features. However, because iatrogenic wounds are caused by medical activities, patients are often reluctant to cooperate or psychologically fear and are reluctant to accept more traumatic treatments. Medical staff must pay attention to the psychological treatment of patients to avoid complaints and emotional disturbance. Thus, the treatment of iatrogenic wounds while avoiding secondary injuries is a challenge for medical staff.

The pathway of healing is determined by characteristics of the wound on initial presentation, and it is vital to select the appropriate method to treat the wound based on its ability to avoid hypoxia, infection, excessive edema, and foreign bodies [47]. It is relatively simple to treat wounds that are unavoidable, such as wounds after laser treatment and donor site wounds after skin graft removal. These wounds should be kept clean and dry, and steps must be taken to reduce exudation and prevent infection; with these measures, most of these wounds heal without complications. For simple wounds, infection should be controlled to prevent wound deepening; most of these wounds heal in 1 to 2 weeks. For complex or refractory wounds, it is necessary to choose a comprehensive treatment based on the condition of the wound. These wounds may require various treatment strategies, including nutritional support, exogenous growth factors, chitosan, hyperbaric oxygen, platelet concentrate, exogenous alginate or biological dressings, debridement, and surgery [48, 49].

Since wounds caused by SSIs account for a large proportion of iatrogenic wounds, guidelines have been developed for the prevention and treatment of SSIs. The treatment of SSIs includes a variety of comprehensive treatments such as pre-hospital interventions, hospital interventions, and post-discharge incision care [50].

In the case of non-iatrogenic wounds, any foreign bodies present within the wound must be removed. Similarly, in the case of implant-related iatrogenic wounds, the implant should be removed and then replaced 4 to 6 months later. However, in some cases, the implant is expensive or essential to the patient, such as pacemakers, silicone breast implants, artificial vascular grafts, and periprosthetic joints [51, 52]. In such cases, salvage treatment can be performed, and if necessary, a salvage operation should be performed to preserve the implants as much as possible and minimize the damage to the patient. Debridement and prosthesis retention may bring good quality of life outcomes to patients and reduce costs [53]. Byren et al. [54] showed that the success rate of 112 infected arthroplasties treated with debridement, antibiotics, and implant retention was 81%. A systematic literature review by Maillet et al. reported that debridement and prosthesis retention in association with prolonged antimicrobial treatment may be an advantageous alternative to arthroplasty exchange for frail patients [55].

**Fig. 3:**
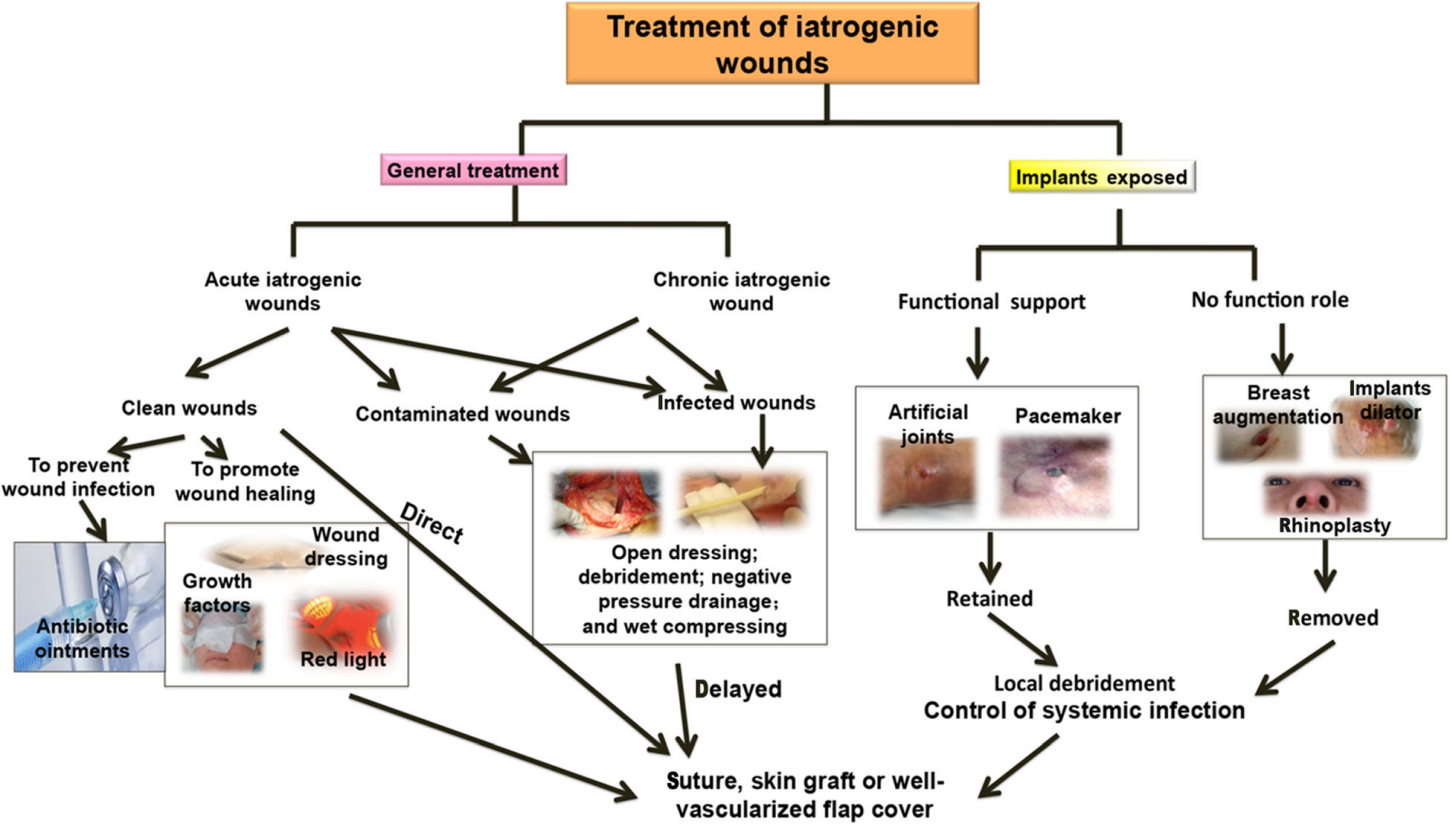
Flowchart of iatrogenic wound treatment includes general treatments and treatments for implant exposure

A review of the literature showed that the treatment of implant-related iatrogenic wounds usually includes the following [44, 52, 56–58] (Fig. 3): (1) the control of systemic infection; (2) local debridement to remove necrotic tissue; (3) wound cleaning and debridement to retain implants, followed by repeated rinsing with a high-pressure washing gun, hydrogen peroxide, and saline, and finally negative-pressure wound therapy; and (4) a well-vascularized myocutaneous flap to cover the wound. When no suitable tissue is present around the wound, the prosthesis can be enclosed with a capsule. Secondary closure of these wounds is usually successful in patients with no related systemic diseases, and sufficient and well-vascularized soft tissue coverage. Successful salvage of ophthalmic and breast implants in patients with infected wounds has been achieved using the above method in China and other countries [57, 58]. Postoperative observation is necessary for the prevention and control of hematoma, infection, and skin flap necrosis. Additionally, proper management is indispensable for tetanus-prone wounds. However, avoiding the recurrence of implant infection is difficult, and implant removal is inevitable in some cases. Vacuum sealing drainage may be applied to enable subsequent wound coverage with a skin graft or skin flap.

## Conclusion

Iatrogenic wounds are a common problem with unique features. Medical staff must be better educated on medical ethics and improve their medical knowledge to avoid the occurrence of avoidable iatrogenic wounds. If iatrogenic wounds do occur, efforts must be made to accelerate wound healing as soon as possible while avoiding secondary injuries.

## Abbreviations

SSI: Surgical site infection

## Authors’ information

Biao Cheng, MD, PhD, Professor, is a professor of surgery at Wound Healing Unit, Department of Plastic Surgery, General Hospital of Southern Theater Command of PLA. His research interests include tissue regeneration and repair.

Ju Tian, MD, is a surgeon at Department of Plastic Surgery, People’s Hospital of Zhongshan City. His research interests include tissue regeneration and repair.

Yan Peng, MD, is a surgeon-scientist at the Department of Orthopaedics and Traumatology, Li Ka Shing Faculty of Medicine, The University of Hong Kong. Her research interests include tissue regeneration and repair.

Xiaobing Fu, MD, PhD, Professor, is a member of China Engineering Academy and a professor at Wound Healing Unit, the First Affiliated Hospital, General Hospital of PLA, Medical College of PLA. His research interests include trauma medical research and tissue regeneration and repair.
